# Supplementary material for: Self‐Solidifying Active Droplets Showing Memory‐Induced Chirality
Source: Adv Sci (Weinh). 2023 Aug 1;10(27):2300866. doi: 10.1002/advs.202300866 (PMC10520641; doi:10.1002/advs.202300866)
Supplement: Supplementary file 1 — Supporting Information [file ADVS-10-2300866-s003.pdf]

ADVANCED SCIENCE
Open Access

Supporting Information

for *Adv. Sci.*, DOI 10.1002/advs.202300866

Self-Solidifying Active Droplets Showing Memory-Induced Chirality

Kai Feng, José Carlos Ureña Marcos, Aritra K. Mukhopadhyay, Ran Niu, Qiang Zhao, Jinping Qu and Benno Liebchen*

Self-solidifying active droplets showing memory-induced chirality

Kai Feng[1], José Carlos Ureña Marcos[2], Aritra K. Mukhopadhyay[2], Ran Niu[1,✉], Qiang Zhao[1], Jinping Qu[1] & Benno Liebchen[2]

June 28, 2023

Supplementary Note 1: pH-induced aqueous phase separation

We explored the influence of polyelectrolyte concentration on the motion of the droplet. Droplets of concentration $\leq$ 10 wt% cannot form a stable water-droplet interface and instead collapse when dipped on the air-water interface. Due to the high molecular weight of PSS and PEI, it is hard to get a uniform solution when the polyelectrolyte concentration is higher than 40 wt%. Therefore, droplets of concentrations of 15 wt%, 25 wt% and 35 wt% were prepared. As shown in Supplementary Fig. 1a, the peak velocity of the droplet increases as the concentration of polyelectrolyte decreases, possibly due to the fast diffusion and complexation of polyelectrolytes in a loose network, which also leads to a shorter self-propulsion time and moving distance of the droplet (Supplementary Fig. 1b). Therefore, we used a droplet of 25 wt% polyelectrolyte concentration for most of the experiments.

The release of PSS molecules into the acidic water solution was confirmed by the UV-Vis absorption spectra of the acidic solution after placing a PEI/PSS droplet on it for 1.5 h. As shown in Supplementary Fig. 2, both pure PSS solutions of different concentrations (0.08–0.40 mg mL^{-1}) and the acidic water solution after the droplet swimmer has completely solidified show a characteristic peak at 262 nm, corresponding to the vibration of the benzene rings.

The dependence of the PSS weight loss on the molar ratio of PSS to PEI was explored. As shown in Supplementary Fig. 3a, the released mass of PSS is lowest at a PSS/PEI molar ratio of 1:1, and increases as the molar ratio increases or decreases. This observation is reasonable as complexation and release are two competing processes, and complexation is predominant at a molar ratio of 1:1. The molar ratio of PSS to PEI also influences the self-propulsion behavior of the droplet swimmer. As shown in Supplementary Fig. 3b, the peak velocity of the droplet slightly increases as the molar ratio of PSS to PEI increases, while the self-propulsion time dramatically decreases as the molar ratio of PSS

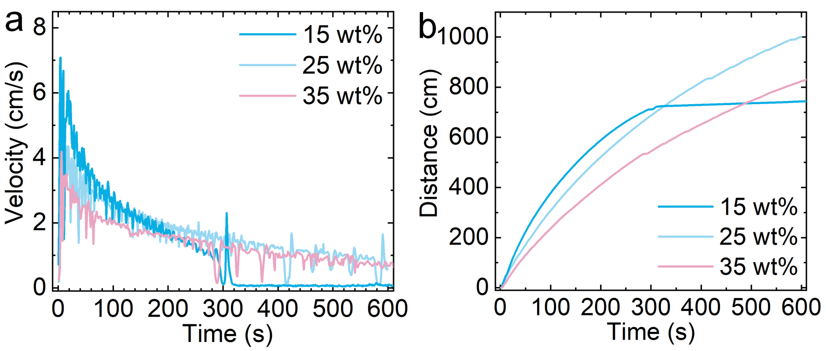

Supplementary Figure 1: Exemplary measurements of (a) the speed and (b) the distance covered by PSS/PEI droplets (molar ratio = 2:1) of different solids contents.

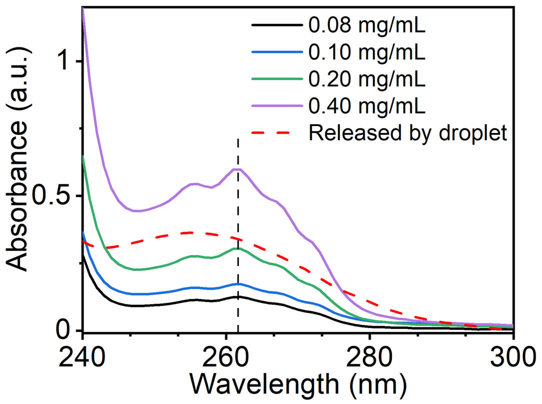

Supplementary Figure 2: UV-Vis spectra of several PSS solutions of different concentrations and the acidic water solution after the droplet swimmer has completely solidified.

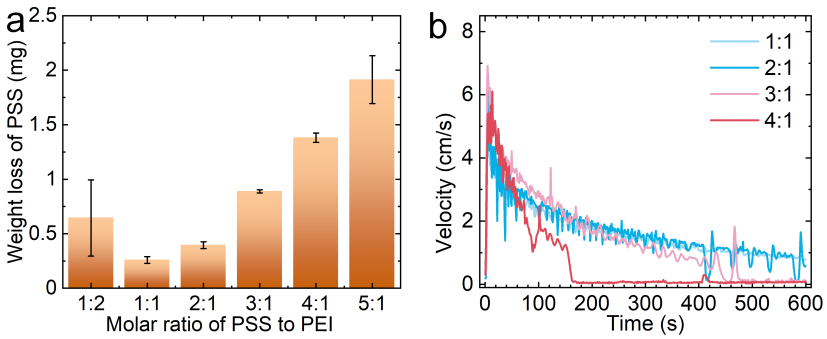

Supplementary Figure 3: (a) Weight loss of PSS for droplets of different PSS-to-PEI molar ratios.(b) Exemplary measurements of the droplet speed for different PSS-to-PEI molar ratios.

to PEI increases. This could be explained by the fast and massive release of PSS, which induces the quick establishment of a surface tension gradient and fast consumption of the fuel at a higher PSS to PEI ratio.

The influence of the specific confining geometry on the motion of the droplet swimmer was further explored in a tank of sides 100 × 60 cm (Supplementary Fig. 4).

We explored the generality of PSS-induced motion by embedding PSS molecules into a gelatin hydrogel. Supplementary Fig. 5 shows the typical velocity and trajectory of the hydrogel swimmer.

The influence of the molecular weight of PSS on the motion of the PEI/PSS droplet is shown in Supplementary Fig. 6. A droplet of PSS of molecular weight 70,000 shows a similar velocity to that of PSS of molecular weight 1,000,000 (Supplementary Fig. 6a). However, the droplet of low PSS molecular weight does not show chiral motion (Supplementary Fig. 6b).

We measured the concentration of PSS at different positions relative to a fixed droplet pump (schematic in Supplementary Fig. 7a). As shown in Supplementary Fig. 7b, the PSS concentration near the droplet decreases with time, whereas the concentration at radial distances of 1.0 cm and 1.5 cm increases with time. The released PSS generates a surface tension gradient at the air-water interface (Supplementary Fig. 7c), as measured with a force tensiometer using a thin plate geometry of 10 mm in width and 5 mm in height. We also characterized the flow profile around the droplet by tracer velocimetry. This is three-dimensional (Supplementary Fig. 7d), as expected due to the incompressibility of the acidic water solution.

We determined the mean starting time of chiral motion to be 282.6 s (Supplementary Fig. 8).

The viscosity of the PSS/PEI solution was measured on a MCR302 rheometer (Anton Paar M302) using the concentric cylinder geometry in the shear rate range of 10^{-2}–10^{2} s^{-1}. As shown in Supplementary Fig. 9, the viscosity of the

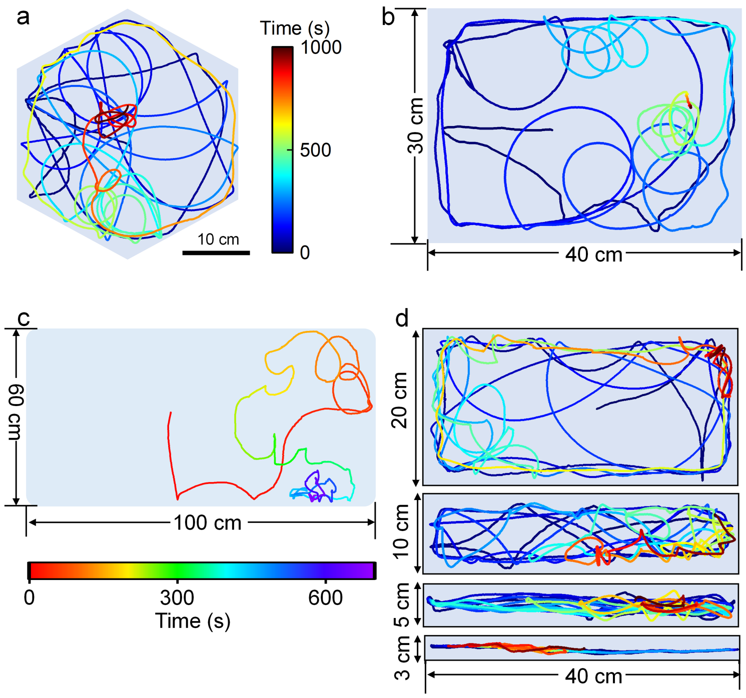

Supplementary Figure 4: Droplet trajectories in (a) a hexagonal vessel (side 20 cm), (b) a rectangular vessel (sides 40 × 30 cm), (c) a tank (sides 100 × 60 cm) and (d) vessels of length 40 cm and different widths.

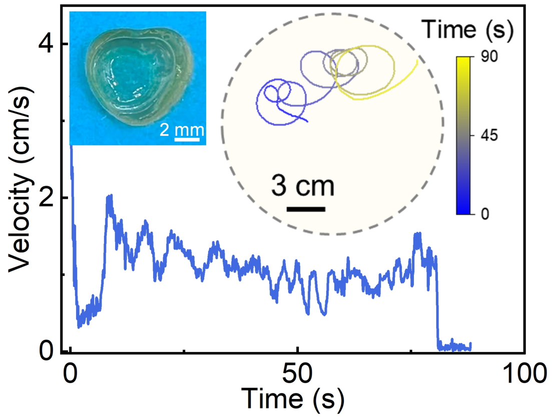

Supplementary Figure 5: Velocity of a gelatin hydrogel with embedded PSS molecules. Insets: photograph of the heart-shaped hydrogel (left) and trajectory of the swimmer (right). The scale bar length is 2 mm.

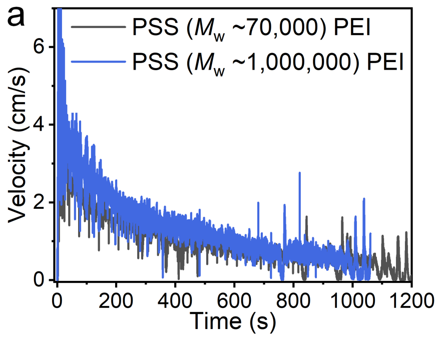

Supplementary Figure 6: (a) Velocity of droplet swimmers composed of PSS of different molecular weights. (b) Trajectory of the droplet swimmer of PSS molecular weight of 70,000.

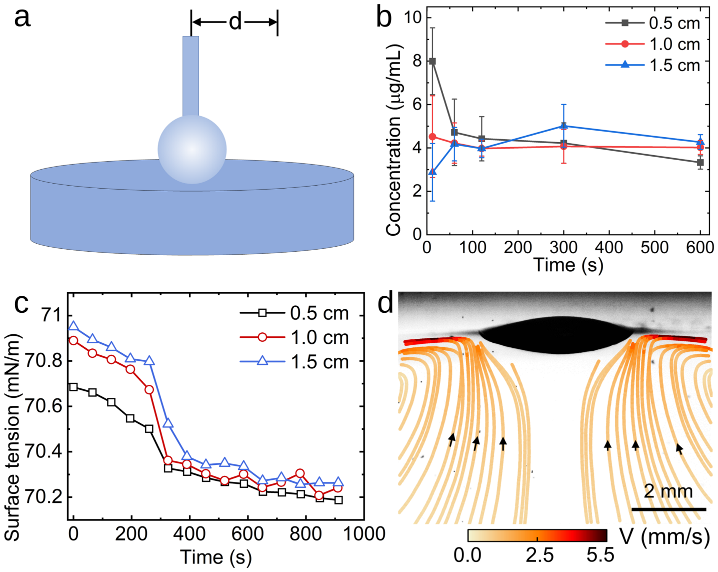

Supplementary Figure 7: (a) Schematic of the fixed droplet pump. (b) Time evolution of the PSS concentration and (c) the surface tension at the air-water interface at different positions relative to the center of the fixed droplet. (d) Side view of the fluid streamlines around the fixed droplet.

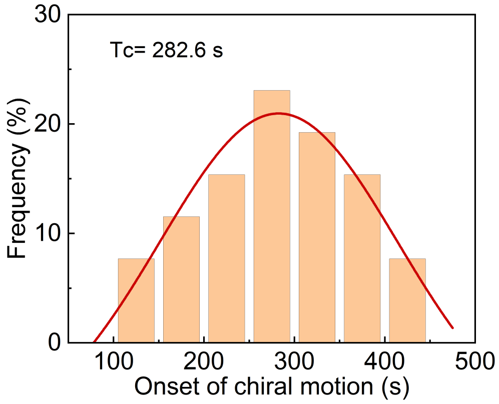

Supplementary Figure 8: Distribution of the starting time of chiral motion in 30 experimental runs in a circular Petri dish of radius $R = 10$ cm. The mean starting time is $T_c = 282.6$ s.

PSS/PEI solution is much higher than that of water.

Supplementary Note 2: Comment on the used model

The droplet is initially liquid. Therefore, rather than considering a phoretic mechanism as usually done when modelling solid swimmers, a stress jump at a fluid-fluid interface is considered here to model the onset of motion. The droplet indeed solidifies with time, but the solid layer is thin for the considered time scales. Since the inside of the droplet remains liquid and the solid shell is porous (and therefore fluid can still diffuse out of the droplet), we expect a similar fluid flow inside our droplet to that of a purely liquid droplet. At very late times, when the solidified layer is thick, the situation might become much more complex and is beyond the scope of this work.

Supplementary Note 3: Estimation of simulation parameters

A: Diffusion coefficient

The diffusion coefficient D used in our simulations was estimated from the experimental values of the diffusion coefficients of PSS ($D_{\mathrm{PSS}} = 8.74 \pm 0.12$ μm^2 s^{-1} in 80 mM HCl) and PEI ($D_{\mathrm{PEI}} = 6.85 \pm 0.74$ μm^2 s^{-1} in 80 mM HCl). We used the formula $D = N_{\mathrm{PSS}} D_{\mathrm{PSS}} + N_{\mathrm{PEI}} D_{\mathrm{PEI}}$ [1,2], where N_{PSS} and N_{PEI} denote the molar fraction of the corresponding polyelectrolyte. Using the molar

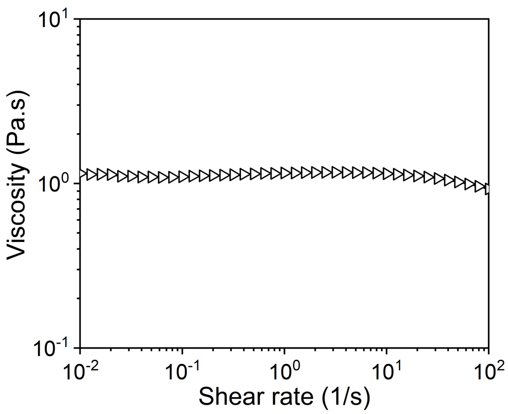

Supplementary Figure 9: Viscosity of the PSS/PEI solution versus shear rate.

ratio of the PEI/PSS solution from the experiments (0.174:0.826), we estimated $D \sim 8\ \mu\text{m}^2\ \text{s}^{-1}$.

B: Polymer production rate

The polymer production rate was chosen by fitting the experimental release rate and total released mass of PSS to exponential time evolutions. We chose $\tau^{-1} \approx 0.11\ \text{min}^{-1}$ as the average value of the parameter b in the fits shown in Fig. 2b (main text) and Supplementary Fig. 10 (see captions for the definition of the fitting parameters a and b). Given the molecular mass of PSS ($M_{\text{PSS}} = 10^6\ \text{g mol}^{-1}$), the initial activity was estimated as $A = \frac{a}{\pi r_{\text{d}}^2 M_{\text{PSS}}} \sim 10^{-7}\ \text{mol m}^{-2}\text{s}^{-1}$. We assumed that, effectively, emission of PSS takes place through a circle of radius r_{d} corresponding to the radius of the droplet swimmer. $A = \frac{2}{3} \times 10^{-7}\ \text{mol m}^{-2}\text{s}^{-1}$ was used in our simulations.

Supplementary Note 4: Mesh independence study

To study the independence of our simulation results on the chosen mesh, we considered the chemical concentration relative to a fixed droplet for highly diffusive polymer molecules ($D = 8 \times 10^{-2}\text{m}^2/\text{s}$), for which the concentration field, the flow field and the droplet motion are weakly coupled. In Supplementary Fig. 11a,b, we can see that all the considered meshes lead to very similar results. The results for the two finest meshes (black and yellow lines) are essentially on top of each other. Their relative error (Supplementary Fig. 11b), integrated over the shown distance regime, amounts to only 0.0059 percent. Even the difference between the finest and the roughest mesh leads to an integrated relative error of only 0.0194 percent.

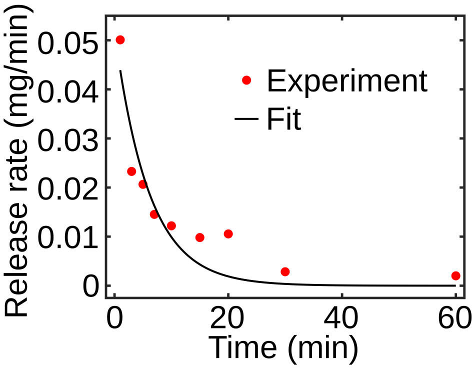

Supplementary Figure 10: Time evolution of the release rate of PSS by the droplet. The black line corresponds to a fit to ae^{-bt}, which yields $a = 0.052$ mg min^{-1} and $b = 0.165$ min^{-1}. The experimental data were obtained by measuring the concentration of PSS released by the droplet in 30 mL of acidic water solution in 10 independent measurements. The acidic solution was carefully stirred by hand to ensure a homogeneous distribution of PSS before taking each specimen (0.5 mL). The release rate is the time derivative of the released mass in Fig. 2b (main text).

Additionally, we explicitly tested the mesh dependence of our results in parameter regimes where the concentration field, the flow field and the droplet motion are strongly coupled. Here, the droplet speed (Fig. 3d in the main text) sensitively depends on the initial conditions (and on numerical noise) and fluctuates from simulation to simulation, even for one and the same mesh (similar to what is observed in experiments, where the trajectory and the droplet speed also fluctuate when repeating the experiment under identical conditions). Accordingly, we simulated the time evolution of the droplet speed for two different meshes (the one used in the main text and a much finer one) and averaged the result over 85 individual runs for each mesh. As shown in Supplementary Fig. 11c, the results agree within error bars (error bars show the standard deviation of the speed for one and the same mesh). The peak position and the peak height are almost identical for the two meshes. In addition, we tested the normalized and radially averaged flow speed as a function of distance from the droplet surface (Fig. 3c in the main text) for different meshes. These results also agree well with each other.

In the main text, we chose the following meshes:

- Figs. 3c, 4, 5, 6 and 8: mesh 1 in Supplementary Fig. 11a (which is the same mesh as mesh 1 in the inset of Supplementary Fig. 11c).
- Fig. 7: mesh 3 in Supplementary Fig. 11a.
- Fig. 3d: mesh 1 in the main plot of Supplementary Fig. 11c.

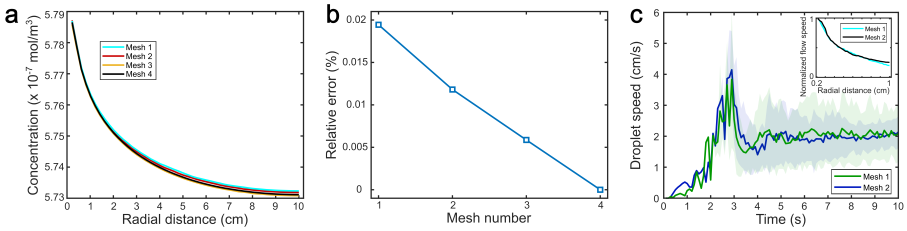

Supplementary Figure 11: Mesh independence study. (a) Average chemical concentration relative to a fixed droplet for highly diffusive polymer molecules ($D = 8 \times 10^{-2} \mathrm{m}^2/\mathrm{s}$) after 20 s. (b) Integrated error relative to mesh 4 for each mesh in panel a. (c) Droplet speed far from any boundaries (see Fig. 3d in the main text; averaged over 85 runs per mesh) with shaded error bars representing the standard deviation. Note that all trajectories corresponding to each mesh are almost identical until about 2 s, and so there are no visible error bars up to that time. Inset: Average flow speed relative to the fixed droplet used in Fig. 3c in the main text after 10 s (averaged over 20 runs per mesh). Number of elements in a and b (from mesh 1 to mesh 4): 5602, 6726, 10642, 73656. Number of elements in c (main plot): 4022, 31524. Number of elements in c (inset): 5602, 73656.

Supplementary Note 5: Dependence of the droplet speed on polyelectrolyte concentration

We performed simulations with two different PSS release rates, matched with the experimentally measured ones. Here, we considered (i) PSS-concentration dependent droplet sizes, as in experiments (r_d=0.22 and 0.27 cm for droplets of solids content 25 $wt\%$ and 35 $wt\%$, respectively), and (ii) droplets of identical size. In case (i), Supplementary Fig. 12a, we see that the droplet velocity is lower for higher PSS concentration, as in experiments. In case (ii), Supplementary Fig. 12b, we see the opposite effect, i.e. a higher droplet speed for higher PSS concentration. From this, we conclude that higher release rates lead to higher velocities, as expected. However, in our experiments this effect is overcompensated by the dependence of the droplet speed on the droplet size. In the experiments, the droplet size is influenced by the intrinsic properties of the droplet, such as viscosity, surface tension and density, and so it is hard to control when added into acidic water with a pipette.

Supplementary Note 6: Spontaneous symmetry breaking triggers self-propulsion

In order to support our claim that droplet self-propulsion in our system is induced by spontaneous symmetry breaking, we ran simulations varying the diffu-

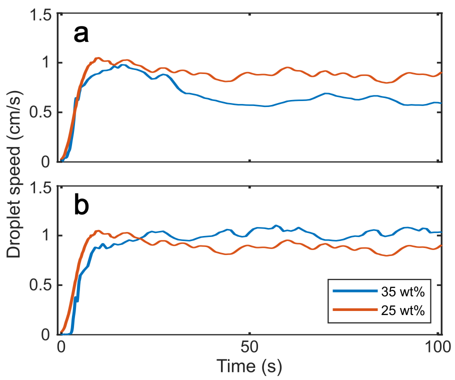

Supplementary Figure 12: Droplet speed (moving mean, averaged over 5 s) for different PSS concentrations inside the droplet. (a) Droplet size as in Supplementary Fig. 1. (b) Fixed droplet radius: $r_{\mathrm{d}} = 0.22$ cm.

sion coefficient of the emitted polymer molecules. As shown in Supplementary Fig. 13, the droplet only self-propels for low enough values of the diffusion coefficient, or in other words, for high enough Péclet numbers, as expected for droplet swimmers self-propelling as a result of spontaneous symmetry breaking (Refs. [53,54] in the main text).

Supplementary Note 7: Vorticity and concentration profiles

The vorticity of the fluid around the droplet remains approximately constant as long as the droplet moves ballistically (Supplementary Fig. 14a). Contrastingly, in the chiral regime we observe the repetitive pattern 1-2-3-4 shown in Supplementary Fig. 14b-e, and the corresponding oscillations in the value of the average vorticity (Fig. 8c in the main text). Our simulations unveil the relationship between the polymeric trail of the droplet and the fluid vorticity in the water domain and, in particular, in the surroundings of the droplet (Supplementary Fig. 14f-j), thus illustrating the importance of memory effects in the advent of chirality in our system.

Supplementary Note 8: Setup for self-propulsion in weak acidic water

The dimensions of the two-chamber cell used to achieve droplet self-propulsion in weak acidic water is shown in Supplementary Fig. 15.

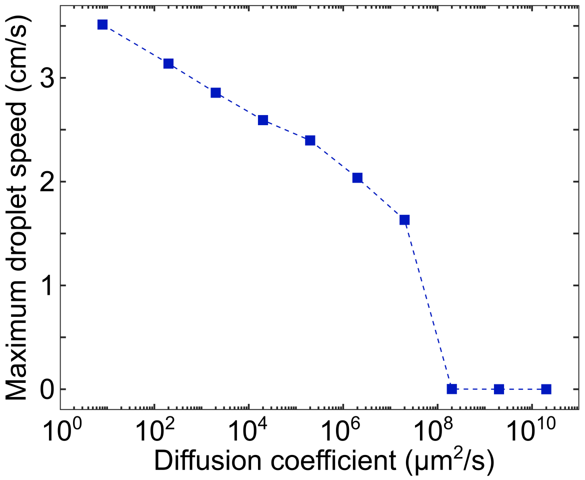

Supplementary Figure 13: Maximum droplet speed as a function of the diffusion coefficient of the emitted polymer molecules in the simulations. Simulation details as in Fig. 3d in the main text.

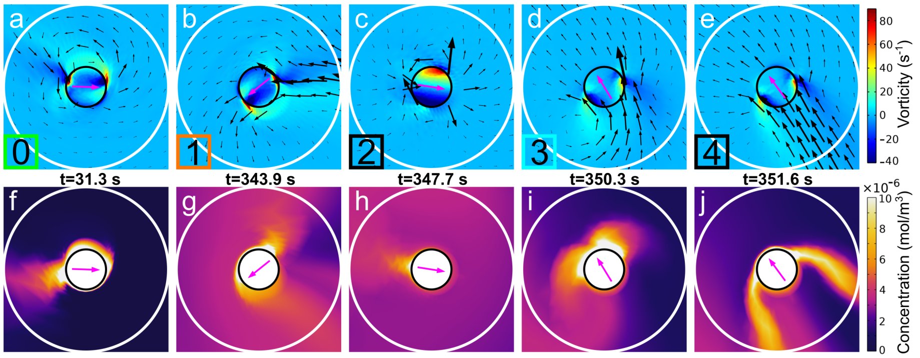

Supplementary Figure 14: Vorticity (a–e) and concentration (f–j) profiles at the representative instants (0–4) of droplet motion sketched in Fig. 6g (see main text). The length of the black arrows in (a–e) is proportional to the flow speed. The magenta arrows indicate the direction of droplet motion. The average vorticity Ω is calculated within the annular region between the droplet boundary and the white circle (see 'Methods' in the main text).

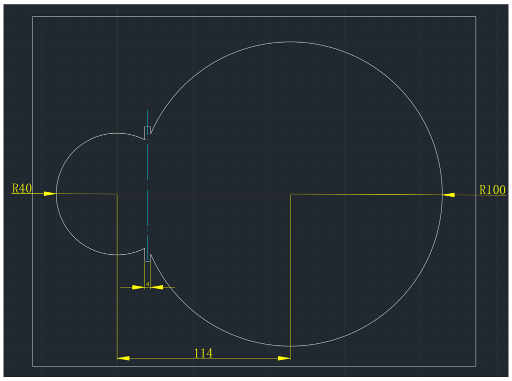

Supplementary Figure 15: Blueprint for the two-chamber cell used to achieve self-propulsion in weak acidic water. Dimensions in mm.

Supplementary Note 9: Method of measuring uranium element concentration

We used arsenazo (III) as a uranium reagent, which can coordinate with the uranyl ions in aqueous solutions. The formed complex was detected in the UV-Vis spectra of the uranium solution, where a specific absorption peak appears at 650 nm and the absorbance is linear with the concentration of uranyl ions in a certain range (Supplementary Fig. 16). Fifteen droplets complexed for different times were added into a uranium solution of 10 ppm and pH 4.3. At set time intervals, 100 μL of solution were taken and passed through a 0.45 μm filter. After adding 100 μL of 0.1 mol/L HCl, 200 μL of 0.5 mg/mL arsenazo (III) aqueous solution and 600 μL of ultrapure water into the test solution, the absorbance was measured at a fixed wavelength of 650 nm with a UV-Vis spectrophotometer. The uranium concentration was then determined according to Supplementary Fig. 16.

In an external PSS field generated by a PSS-releasing hydrogel, the droplet avoids the high-concentration region in the first 90 s (Supplementary Fig. 17).

The removal performance of the droplet for different volumes and concentrations of uranium waste was investigated. The removal efficiency decreases from 84.7% to 47.4% as the volume of uranium waste increases from 10 to 100 mL (Supplementary Fig. 19c). Moreover, as the concentration of uranium increases from 10 mg/L to 100 mg/L, the removal efficiency decreases from 84.7% to 35.2% (Supplementary Fig. 19d), due to the limited number of adsorption sites on the droplet.

We investigated the influence of the molar ratio of PSS to PEI on the uranium adsorption performance of the droplet swimmer. As shown in Supplementary Fig. 20, the uranium removal efficiency of the droplet increases from 21.7% to 84.7%, 90.4% and 93.9% as the molar ratio of PSS to PEI increases from 1:1 to

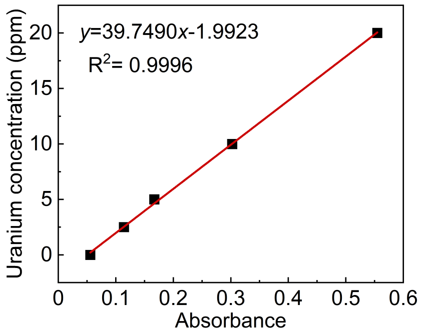

Supplementary Figure 16: Linear regression of the uranium concentration as a function of absorbance in waste water.

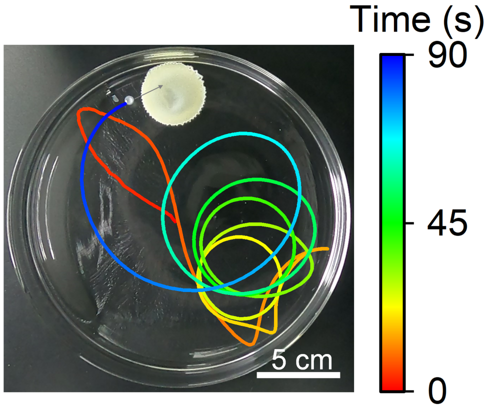

Supplementary Figure 17: Droplet trajectory in an external PSS field generated by a PSS-releasing hydrogel.

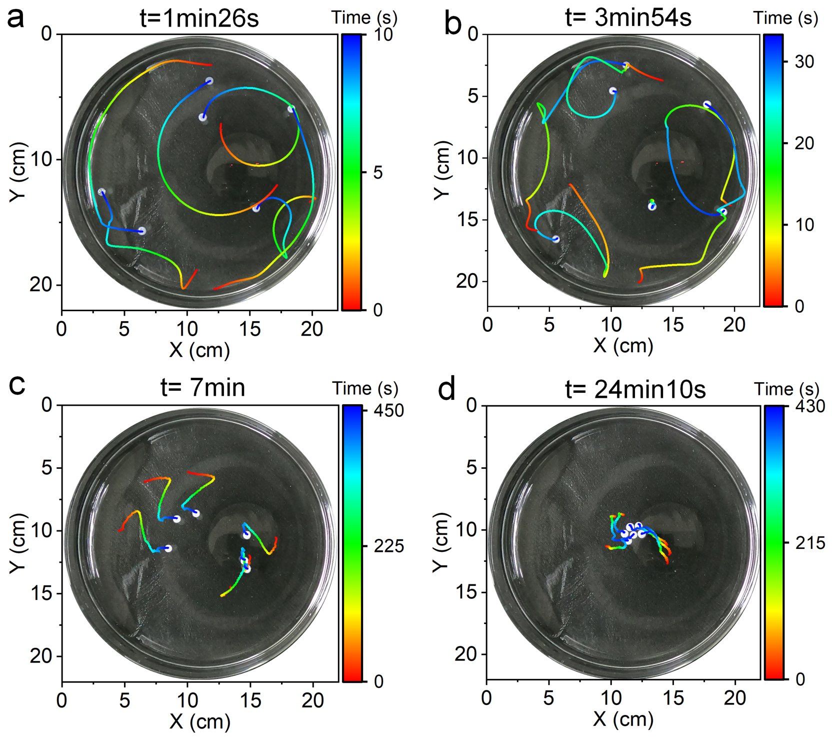

Supplementary Figure 18: Trajectories of six droplets in a circular Petri dish (diameter 20 cm).

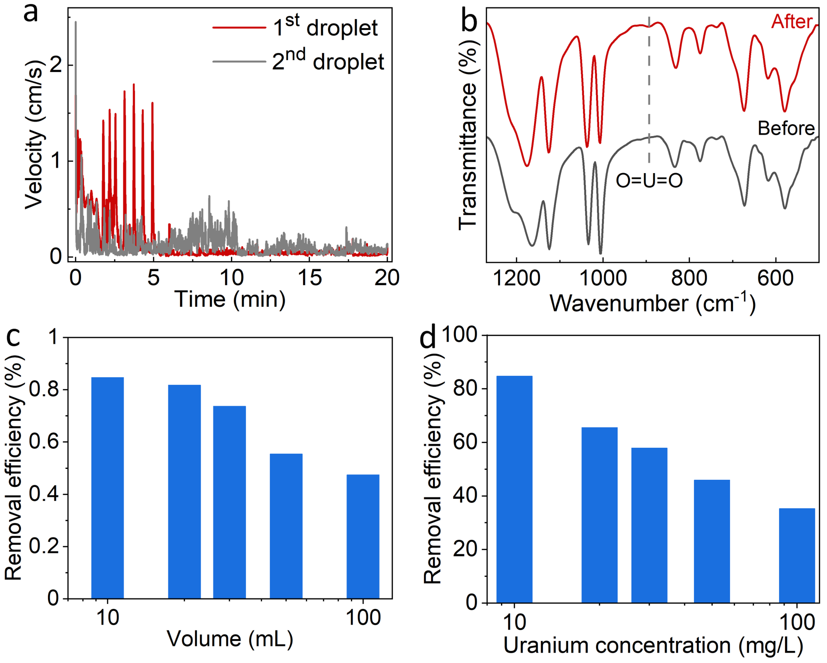

Supplementary Figure 19: (a) Velocity of the 1st and 2nd droplets in a uranium solution of 10 ppm. (b) FTIR spectra of the 1st droplet swimmer before and after adsorption of uranium. Uranium removal efficiency of the 1st droplet in a uranium solution of (c) different volumes (10 ppm) and (d) different concentrations (10 mL).

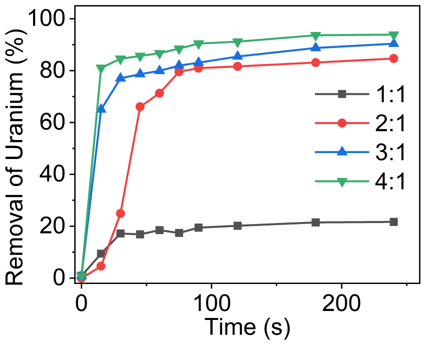

Supplementary Figure 20: Uranium removal kinetics for 1st droplets (see main text) of different PSS-to-PEI molar ratios.

2:1, 3:1 and 4:1, thanks to sulfonate-uranyl coordination.

Supplementary Note 10: Description of Supplementary Movies

Movie S1: Experiment showing ballistic droplet dynamics. Experimental conditions as described in 'Methods' (main text). Playback speed is 20x real-time speed.

Movie S2: Experiment showing chiral droplet dynamics. Experimental conditions as described in 'Methods' (main text). Playback speed is 20x real-time speed.

Movie S3: Experiment showing self-trapping droplet dynamics. Experimental conditions as described in 'Methods' (main text). Playback speed is 20x real-time speed.

Movie S4: Experiment showing that a droplet with a low PSS release rate does not self-propel. The droplet first solidified in acidic water for 15 min and was then moved to another Petri dish with acidic water. Playback speed is 10x real-time speed.

Movie S5: Simulation of the droplet dynamics (left, concentration profile; right, flow field). See 'Methods' in the main text for the simulation details.

Movie S6: Simulation showing ballistic droplet motion in a circular high concentration domain. See 'Methods' in the main text for the simulation details.

Movie S7: Simulation showing chiral droplet motion in a circular high concentration domain. See 'Methods' in the main text for the simulation details.

Movie S8: Experiment showing the collective behavior of six droplets in a circular Petri dish (diameter 20 cm). Playback speed is 10x real-time speed.

References

[1] G. S. Hartley, and J. Crank, Some fundamental definitions and concepts in diffusion processes, *Trans. Faraday Soc.* **45**, 801–818 (1949).

[2] A. Vignes, Diffusion in Binary Solutions. Variation of Diffusion Coefficient with Composition, *Ind. Eng. Chem. Fundam.* **5**, 189–199 (1966).
